# High immune efficacy against different avian influenza H5N1 viruses due to oral administration of a *Saccharomyces cerevisiae*-based vaccine in chickens

**DOI:** 10.1038/s41598-021-88413-2

**Published:** 2021-04-26

**Authors:** Han Lei, Xin Lu, Shuangqin Li, Yi Ren

**Affiliations:** College of Medicine, Southwest Jiaotong University, Chengdu, Sichuan China

**Keywords:** Biotechnology, Expression systems, Protein delivery, Immunology, Vaccines

## Abstract

A safe and effective vaccine is the best way to control large-scale highly pathogenic avian influenza virus (HPAI) A (H5N1) outbreaks. *Saccharomyces cerevisiae* (*S. cerevisiae*) is an ideal mucosal delivery vector for vaccine development, and we have previously shown that conventional administration of a *S. cerevisiae*-based vaccine (EBY100/pYD1-HA) via injection led to protection against the homologous H5N1 virus in a mouse model. Because the diameter of *S. cerevisiae* is approximately 10 μm, which results in a severe inflammation by injection route, therefore, oral administration is a more suitable approach for EBY100/pYD1-HA conferring protection in poultry. We extended our work by evaluating the immunogenicity and protective efficacy of oral vaccination with EBY100/pYD1-HA in the chicken model. Oral immunization with EBY100/pYD1-HA could induce robust serum IgG, mucosal IgA and cellular immune responses. Importantly, EBY100/pYD1-HA provided protection against challenges with a homologous and a heterologous H5N1 viruses. These findings suggest that EBY100/pYD1-HA, a promising H5N1 oral vaccine candidate, can avoid potential reassortment of other avian influenza viruses in oral administration of live virus vaccines and overcome the limitations of conventional injection routes. Importantly, this platform will be able to provide opportunities for broader applications in poultry during HPAI A (H5N1) outbreaks.

## Introduction

The emergence and spread of highly pathogenic avian influenza (HPAI) A (H5N1) viruses have fueled concerns of a potential zoonotic pandemic originating in poultry^1^, and spurred efforts towards developing vaccines against A (H5N1) influenza viruses and improving vaccine production methods^2^. The current licensed vaccines, including adjuvanted formulations, predominately include inactivated whole avian influenza H5N1 and are available for the control of outbreaks in poultry^3^. These vaccines have limitations since they require intramuscular injection, and the biosecurity of these vaccines has not been fully elucidated^4^. In addition, some of these vaccines were poorly immunogenic and may require a higher concentration of the immunogen to achieve protective immunogenicity^5^. Further, the egg-based manufacturing processes of these vaccines also have safety and production issues. A live-attenuated A (H5N1) vaccine has been generated by reverse genetics, but the risk of virus reassortment in the field prohibits the use of this vaccine in most instances^6^. Thus, there is a clear need for new vaccine formulations and delivery strategies that can provide increased efficacy and safety.

In attempts to develop more efficacious A(H5N1) vaccines, several formulations, such as mammalian cell-based vaccines^7^, recombinant protein-based vaccines^8^, recombinant virus-like particle (VLP) vaccines^9^, DNA vaccines^10^, bacteria- or yeast-vectored vaccines^11,12^ and viral-vectored vaccines^13,14^, have been extensively explored as alternative approaches. Included in the list of alternative strategies are the recombinant yeast-based A (H5N1) vaccines, which are promising candidates that meet the requirement of vaccine production in a timely manner and can induce robust protective immunity against A(H5N1) virus infection^15^.

*Saccharomyces cerevisiae* is a representative strain of yeast and is widely used in industries performing fermentation, particularly for the food industry. As a novel strategy in the fight against infectious diseases, *S. cerevisiae*-based vaccines hold great promise for both public health and domestic poultry^16^. Mucosal delivery administration of vaccine is superior to conventional methods of injection in terms of operative ease and safety^17^. Oral administration is an economical approach for enhancing mucosal immunity in order to control influenza virus infection while reducing the cost of vaccine delivery^18^. Furthermore, compared to the intracellular expression of recombinant viral proteins, the display of viral proteins on the carrier cell surface can facilitate their recognition by the host mucosal immune system, thereby enhancing their capability of eliciting protective immunity^17^.

We have previously shown that EBY100/pYD1-HA could provide protection when administered by injection route in a mouse model^15^. However, EBY100/pYD1-HA induces serious inflammation at the injection site due to the diameter of *S. cerevisiae* is approximately 10 μm^19,20^. Furthermore, chickens are the primary model for studies of pathogenicity and vaccine efficacy studies for poultry^21^. To address this issue, we hypothesize that oral vaccination with unadjuvanted EBY100/pYD1-HA can produce protective immunity in the chicken model and can be considered an effective platform for the development of an influenza A (H5N1) vaccine for the mass vaccination of poultry.

In the present study, we extended our previous work by evaluating the immunogenicity of EBY100/pYD1-HA in a chicken model. Oral vaccination with EBY100/pYD1-HA induces strong humoral, cell-mediated and mucosal immunity and confers protection against challenges with a homologous and a heterologous H5N1 viruses. Importantly, the production of EBY100/pYD1-HA just requires 2 weeks, and thus, the vaccine has great potential for mass production in a short period of time for use in poultry during influenza A (H5N1) outbreaks.

## Results

### Expression and quantification of EBY100/pYD1-HA

**Figure 1:**
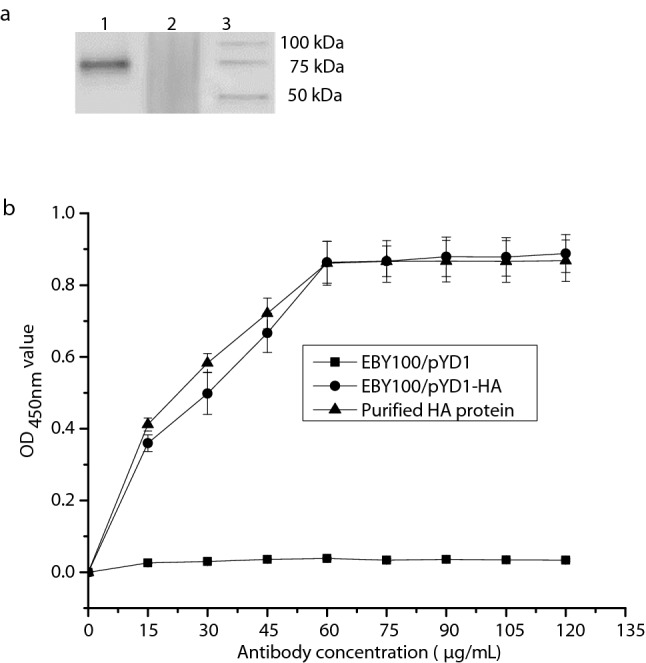
Determinations of expression and quantification of EBY100/pYD1-HA. (**a**) The display of cropped Western blots. Lane 1: EBY100/pYD1-HA; Lane 2: EBY100/pYD1; Lane 3: Western blot marker (Precision Plus Protein™, Bio-Rad). (**b**) Quantification of EBY100/pYD1-HA expressing the HA protein measured by ELISA. The OD_450 nm_ values were obtained from three independent experiments. The bar indicates the means ± SDs. Full-length Western blots are presented in Supplementary Figure 1.

Western blot analysis was performed to determine the expression of HA protein, the expected band corresponding to 75 kDa was observed in the lysates of EBY100/pYD1-HA (Fig. 1a, Lane 1), which consisted of Aga2 (10 kDa) and HA protein (65 kDa), whereas it was absent in the lysates of EBY100/pYD1 (Fig. 1a, Lane 2).

As shown in Fig. 1b, it was found that the concentration of the displayed HA protein was approximately 60 μg/mL on the cell surface of *S. cerevisiae* (Fig. 1b), when increasing concentration of monoclonal anti-HA antibody was used against 5 OD_600nm_ of EBY100/pYD1-HA. When the concentration of antibody was increased beyond this point, the optical density was relatively stable, which suggested that 5 OD_600nm_ of EBY100/pYD1-HA expressing HA protein was at its saturation limit at 60 μg/mL compared with the known concentration of purified HA protein.

### Determination of HA-specific antibody responses

**Figure 2:**
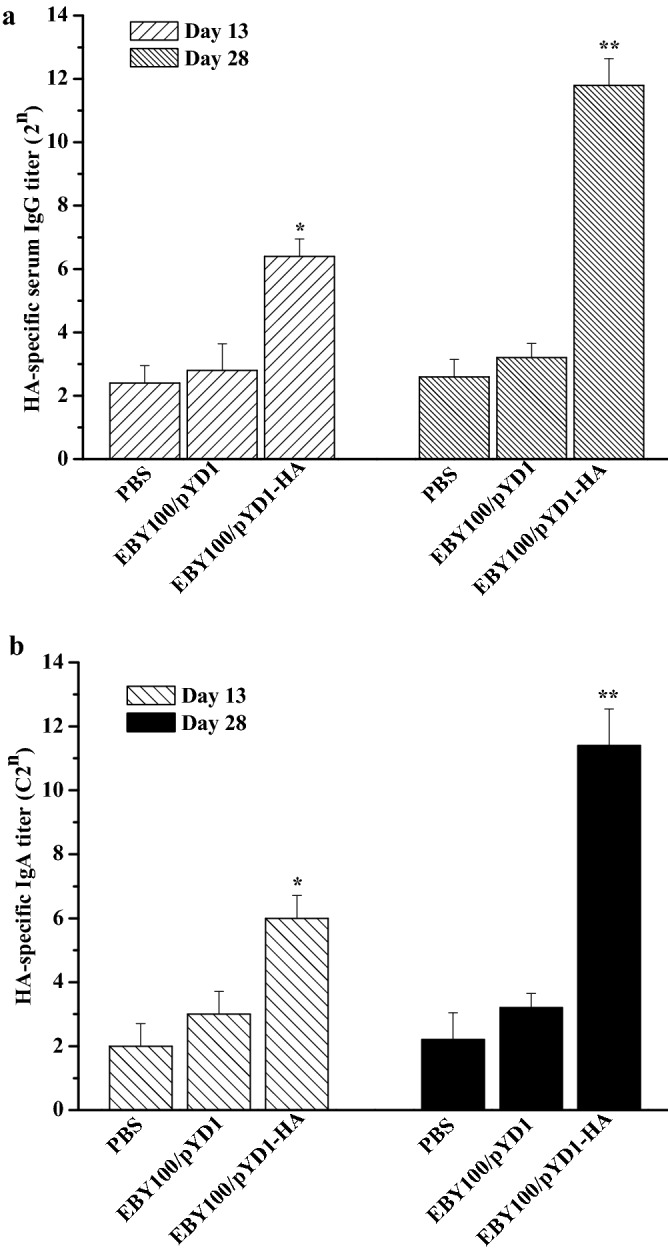
Antibody responses elicited by oral administration of EBY/pYD1-HA. (**a**) HA-specific IgG titer in the serum. (**b**) Secretory mucosal IgA titer in the small intestine washes. Asterisks represent statistically significant differences compare with the PBS- and EBY100/pYD1 controls. *p ˂ 0.05, **p ˂ 0.01.

To evaluate the antibody responses induced by EBY100/pYD1-HA, the IgG levels in the serum and the IgA levels in the intestine washes were separately measured by ELISA on days 13 and 28 after the initial vaccination. The group that received EBY100/pYD1-HA was able to respond with effective and significant HA-specific serum IgG (Fig. 2a) and mucosal IgA antibody (Fig. 2b) levels compared to control groups (PBS and EBY100/pYD1). Therefore, these results indicate that oral administration of EBY100/pYD1-HA can induce robust humoral and mucosal immune responses in a chicken model.

### Cellular immune responses induced by EBY100/pYD1-HA

**Figure 3:**
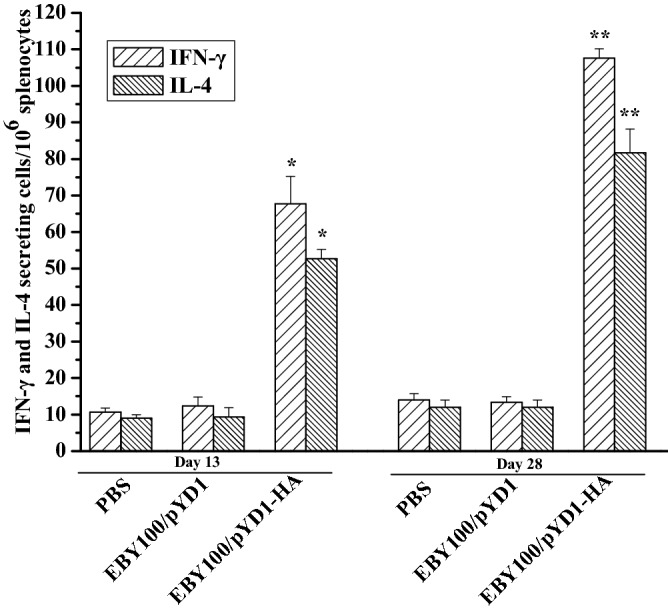
Cellular immune responses induced by oral administration of EBY/pYD1-HA. IFN-γ- and IL-4- secreting cells (n = 5 chickens per group) were separately analyzed by ELISpot assay. Asterisks indicate significant difference compare with the PBS- and EBY100/pYD1 controls. *p ˂ 0.05, **p ˂ 0.01.

To further examine the cellular immunity induced by EBY100/pYD1-HA, we assessed IFN-γ and IL-4-secreting splenocytes using ELISpot kits. Splenocytes were isolated from the vaccinated chickens on days 13 and 28 after the initial immunization and stimulated with a HA-specific peptide. The levels of IFN-γ and IL-4-secreting cells in the EBY100/pYD1-HA group were significantly higher than those in the control groups (Fig. 3). The levels of IFN-γ-secreting cells were higher than the levels of IL-4-secreting cells in the EBY100/pYD1-HA group (Fig. 3). Taken together, these results demonstrate that EBY100/pYD1-HA can induce both Th1- and Th2-type immune responses, with preference of the Th1 type immune responses, as evidenced by the higher levels of IFN-γ production.

### HI titers

To assess the induction of functional antibody responses elicited by EBY100/pYD1-HA, serum was collected from the chickens orally administrated with PBS or EBY100/pYD1. Regardless of doses, these chickens showed only background levels of HI titers. However, EBY100/pYD1-HA could elicit meaningful HI titers of 64 and 64 against A/Vietnam/1203/2004 (H5N1) (clade 1) or A/Chicken/Henan/12/2004 (H5N1) (clade 8), respectively, on days 28 (Table 1). Therefore, EBY100/pYD1-HA is immunogenic and elicits higher levels of functional antibody responses than the PBS- and EBY100/pYD1 controls.

**Table 1:** HI titers.

Groups	A/Vietnam/1203/2004 (H5N1)		A/chicken/Henan/12/2004(H5N1)	
	Day 13	Day 28	Day 13	Day 28
PBS	4	8	8	8
EBY100/pYD1	8	8	8	8
EBY100/pYD1-HA	32*	64**	16*	64**

HI titers are representative of three independent experiments. Asterisks represent statistical significance compared with the PBS- and EBY100/pYD1 groups*.* *p ˂ 0.05, **p ˂ 0.01.

### Immune protective efficacy induced by EBY100/pYD1-HA

**Figure 4:**
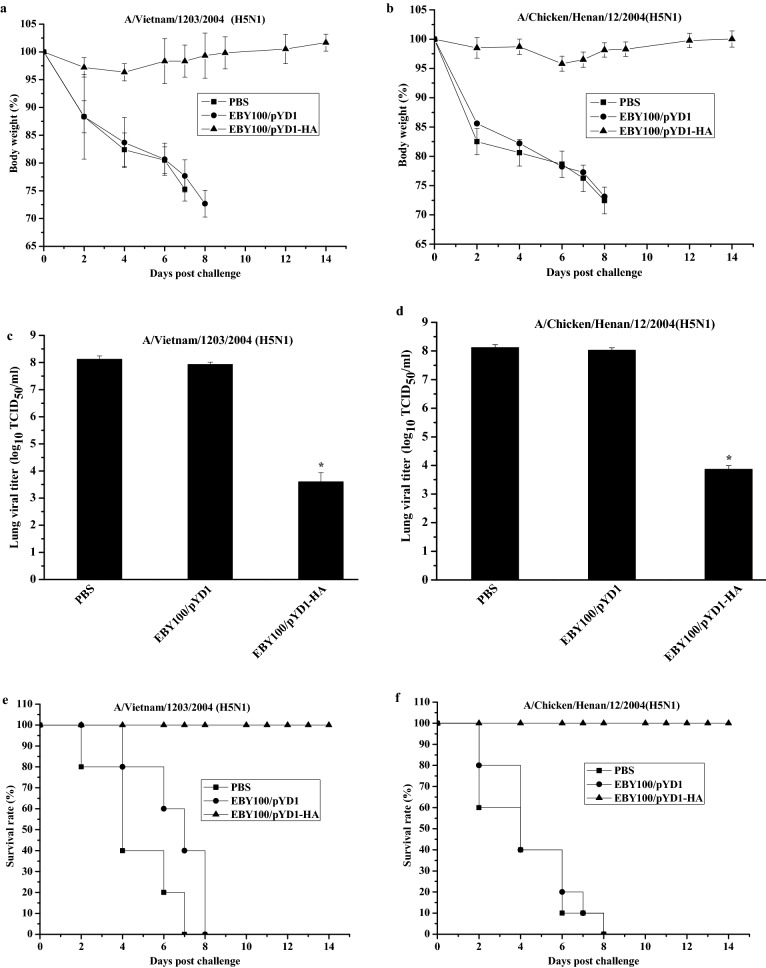
Immune protection efficacy of EBY/pYD1-HA against different H5N1 viruses. Chickens were intranasally challenged with a lethal dose (25 μL of 10^4^ EID_50_) of H5N1 virus. (**a**) Weight change as a percentage after A/Vietnam/1203/2004 (H5N1) (clade 1) challenge. (**b**) Weight change as a percentage after A/Chicken/Henan/12/2004 (H5N1) (clade 8) challenge. (**c**) Lung viral titers at day 3 post A/Vietnam/1203/2004 (H5N1) (clade 1) challenge. (**d**) Lung viral titers at day 3 after A/Chicken/Henan/12/2004(H5N1) (clade 8) challenge. (n = 3 chickens per group). (**e**) Survival rates after A/Vietnam/1203/2004 (H5N1) (clade 1) challenge. (**f**) Survival rates after A/Chicken/Henan/12/2004(H5N1) (clade 8) challenge. (n = 10 per group). The weight change data are presented as the means ± SDs. Asterisks indicate significant difference compared with the PBS- and EBY100/pYD1 controls. (*p ˂ 0.05).

To support the potential of EBY100/pYD1-HA to elicit protective responses against different H5N1 viruses, the vaccinated chickens (n = 16 per group) were challenged with a lethal dose (25 μL of 10^4^ EID_50_) of a homologous A/Vietnam/1203/2004 (H5N1) (clade 1) or a heterologous A/Chicken/Henan/12/2004(H5N1) (clade 8) virus 2 weeks after the final immunization. The conditions of the chickens in terms of changes in body weight and survival rate were monitored daily for 14 days. As shown in Fig. 4, the chickens that orally administrated with PBS or EBY100/pYD1 showed clinical symptoms of severe disease including significant morbidity (as indicated by weight loss) at day 5 (Fig. 4a,b) and high viral titers in lung at day 3 after influenza A (H5N1) virus infection (Fig. 4c,d), and these chickens died from lethal infection by 8 days (Fig. 4e,f). In contrast, EBY100/pYD1-HA group presented with slight weight loss (Fig. 4a,b) and statistically lower virus titer in the lungs (Fig. 4c,d), and all of these chickens survived and recovered completely after the challenge (Fig. 4e,f). These results provide reliable evidence that EBY100/pYD1 is an effective immunogen for conferring protective immunity with high efficacy against highly pathogenic H5N1 avian influenza viruses.

## Discussion

Our laboratory has previously reported the construction and characterization of EBY100/pYD1-HA without quantification of the HA protein. Conventional administration of EBY100/pYD1-HA to mice by injection provided effective immune protection against A (H5N1) virus challenge^15^. Furthermore, we generated another yeast-based H7N9 oral vaccine could also protect mice from influenza A (H7N9) challenge^20^. In the present study, we extend our previous work by investigating the immunogenicity of EBY100/pYD1-HA when administered via the oral route and evaluating the protective immunity of EBY100/pYD1-HA in a chicken model. Our results demonstrate that chickens orally vaccinated with EBY100/pYD1-HA exhibited significantly higher serum IgG and mucosal IgA antibodies responses, higher levels of IFN-γ/IL-4-secreting cells and HI titers. Additionally, the oral administration of EBY100/pYD1-HA in the absence of mucosal adjuvant can confer protection against lethal challenge with a homologous and a heterologous H5N1 viruses. Collectively, these findings highlight the potential of yeast-based vaccine as an alternative approach that represents an important direction for developing an oral vaccine against different H5N1 viruses for use in poultry without the use of injection needles and mucosal adjuvant.

Due to serious limitations of the manufacturing platform, the currently available avian influenza H5N1 vaccines are generally poorly immunogenic and have safety concerns^6,14,22^. Furthermore, most of influenza A (H5N1) vaccines require administration by intramuscular or subcutaneous injection, which has been shown to be insufficient for generating protective immunity at the mucosal surface^23^. The mucosal delivery route can not only induce effective systemic immune responses but also elicit mucosal immune responses, which are helpful for controlling virus replication in the respiratory tract. As an alternative strategy for vaccine development, an edible nonpathogenic vector that carries the recombinant H5N1 HA protein, such as yeast, provides the potential to prevent and control H5N1 virus infection via oral administration.

Oral administration has many advantages over injected parenteral immunization as shown for influenza A (H5N1) vaccines^11^, as well as other pathogens such as rotavirus, typhoid fever in humans^17^. Considering vaccine design, antigen presentation on the surface of yeast is more effective because these antigens are easier to be recognized and presented by M cells in the small intestine^24^. Furthermore, it should be noted that a mucosal delivery system via oral administration of an influenza A (H5N1) vaccine is required to protect the vaccine material from gastric degradation, to promote the uptake of the antigen in the gastrointestinal tract, and to stimulate adaptive immune responses rather than the tolerogenic responses that are stimulated in studies in which soluble antigens are administered^25^. Therefore, the yeast-based vaccine developed here for the oral immunization against different H5N1viruses is an optimal technology for delivering antigens presented on the surface of yeast and represents a future direction of mucosal vaccine development via oral administration.

Regarding the T cell responses, Th1 cells are superior to Th2 cells in providing protection against viral infection by secreting IFN-γ, stimulating B cells, and directing CD8^+^ T cells-mediated cytolysis^26^. The present study supported that the oral administration of EBY100/pYD1-HA was more effective in generating T cells secreting IFN-γ representing Th1-type responses, than in generating IL-4 cytokine, representing Th2-type responses. These observations were also verified by our results from HI assay and virus challenge. It should be noted that the presence of HI antibodies at titers of ≥ 1:40 is considered as an efficacy endpoint^27^. Furthermore, the HA gene used in this study was codon-optimized for completely matching the antigen epitope with a A/Vietnam/1203/2004 (H5N1) (clade 1) and 90% HA protein sequence similarity with a heterologous A/Chicken/Henan/12/2004 (H5N1) (clade 8), which provided the basis of immune protection against a homologous and a heterologous H5N1 viruses.

Virus challenge is regarded as a gold standard method for assessing the immune protective efficacy of an influenza vaccine. The survival rate following influenza virus challenge was more than 60%, highlighting the protective potential of yeast-based vaccine. Compared with the PBS- and EBY100/pYD1 controls, a significant lower lung viral titer in EBY100/pYD1-HA group was detected at day 3 after virus challenge (Fig. 4c,d), but this still may highly suggest that immune schedule of EBY100/pYD1-HA needs to be further optimized for generating a higher neutralizing antibody titer, which may play an important role in reducing virus shedding. Considering the current platforms of approved vaccines, it is important to compare the detailed comparative immunogenicity and protective efficacy of EBY100/pYD1-HA with those of inactivated whole virus and attenuated live H5N1 vaccines. Furthermore, the present study is not a real field study, a field trial is needed to further show the success in real life situation.

In conclusion, the present study evaluated the immune protective efficacy of a *S. cerevisiae* -based H5 vaccine (EBY100/pYD1-HA) in a chicken model. This system can be used as an universal platform for the development of oral vaccines against multiple influenza viruses and offers significant advantages for vaccination, especially in poultry-originated zoonotic infections in humans.

## Materials and methods

### Animals

All experimental protocols involving animals were approved by the ethics committee of Southwest Jiaotong University (Approval number: 7792). All animal procedures were carried out in accordance with the Guidelines for Use and Care of Experimental Animals in Southwest Jiaotong University. All methods are reported in accordance with ARRIVE guidelines.

Specific pathogen-free (SPF) white Leghorn chickens (7 days old) were purchased from SLC Laboratory Center (Shanghai, China) and housed as 5 chicks per cage (50 cm × 45 cm × 45 cm) in an environmentally controlled house. The chickens were fed a pathogen-free diet and water.

All the virus challenge experiments were performed in enhanced animal biosafety level-3 (BSL-3) facilities. Body weight loss of greater than 25% was used as the criterion for euthanasia. All the surviving chickens were euthanized using CO_2_ inhalation for 5 min at 14 days post-infection.

### Vaccine preparedness, oral immunization and sample collection

HA gene (1650 bp) of A/Vietnam/1203/2004 (H5N1) (clade 1) (GenBank accession No. EU122404) without the signal and transmembrane region was subcloned into surface expression plasmid pYD1, EBY100/pYD1-HA was generated as previously described^15^, except that EBY100/pYD1-HA expressing HA was not quantified.

The induction of EBY100/pYD1-HA was expressed in yeast nitrogen base (YNB)—casamino acids (CAA) medium (20 g/L galacotose, 6.7 g/L YNB without amino acids, 13.61 g/L Na_2_HPO_4_, 7.48 g/L NaH_2_PO_4_ and 5 g/L casamino acids) at 20 °C for 72 h.

The expression of HA protein in *S. cerevisiae* was determined by Western blot analysis as described previously^15^. Briefly, 5 OD_600nm_ of EBY100/pYD1-HA pellets were boiled for 10 min with 100 µL of 6× sodium dodecyl sulfate (SDS) loading buffer and then run on a 10% SDS–polyacrylamide gel electrophoresis (SDS-PAGE) gel (Bio-Rad, Hercules, CA, USA). The gel was transferred to a 0.45 μm nitrocellulose membrane. After blocking with 5% nonfat milk at room temperature for 2 h, the membrane was incubated with a monoclonal chicken anti-HA antibody (1:500 diluted) (R&D Systems, USA) overnight at 4 °C, and followed by 1:5000 diluted horseradish peroxidase (HRP)-conjugated goat anti-chicken IgG (R&D Systems, USA) at room temperature for 1 h. Lastly, the membrane was reacted with the West Pico Chemiluminescent Substrate (Bio-Rad, Hercules, CA, USA) in the dark for 5 min and the blot signal was imaged using Molecular Imager ChemiDoc XRS System (Bio-Rad, Hercules, CA, USA). Meanwhile, Precision Plus Protein™ WesternC™ (Bio-Rad, Hercules, CA, USA) was used as a protein marker.

EBY100/pYD1-HA was inactivated at 60 °C for 1 h and then used for subsequent oral immunization. The final concentration of EBY100/pYD1-HA was adjusted to 0.5 optical density (OD)_600 nm_/μL. Phosphate-buffer saline (PBS) and EBY100/pYD1 served as controls.

Three groups of chickens (n = 26 per group) were orally immunized with 200 μL of PBS, 100 OD_600nm_ of EBY100/pYD1 or 100 OD_600nm_ of EBY100/pYD1-HA on day 1 (prime immunization) and day 14 (boost immunization).

Sera (n = 10 per group), intestine washes (n = 5 per group) and spleen (n = 5 per group) were collected from the vaccinated chickens on days 13 and 28 after the initial immunization.

### Quantification of EBY100/pYD1-HA expressing HA protein

Quantification of the HA protein was performed by enzyme-link immunosorbent assay (ELISA) as previously described^20^. In brief, 5 OD_600nm_ of EBY100/pYD1-HA were resuspended in 100 μL of a solution of monoclonal mouse anti-HA antibody (0, 15, 30, 45, 60, 75, 90, 105, 120 μg/mL) (kindly provided by NIH Biodefense and Emerging Infections Research Resources Repository, Manassas, VA, USA) in PBS containing 2% bovine serum albumin (BSA) and incubated at room temperature for 2 h. This was followed by incubation with goat anti-mouse IgG antibody conjugated with horseradish peroxidase (HRP) (1 mg/mL) (R&D Systems, Minneapolis, MN, USA) at room temperature for 1 h. After washing with sterile PBS, the cells were resuspended in 100 µL of the HRP substrate 3,3′,5,5′-tetramethylbenzidine (TMB) (R&D Systems, Minneapolis, MN, USA) in the dark for 25 min, and then, 100 μL of 2 mol/l H_2_SO_4_ was added to stop the reaction. The OD_450nm_ value of the supernatant was measured using a microplate reader. EBY100/pYD1was used a negative control. A solution of purified HA protein (60 µg/mL) (kindly provided by NIH Biodefense and Emerging Infections Research Resources Repository, Manassas, VA, USA) was used a positive control.

### Measurement of antibody responses

HA-specific serum IgG and mucosal IgA antibody levels were separately determined by ELISA as previously described^15^. Briefly, 2 µg of recombinant HA protein of A/Vietnam/1203/2004 (H5N1) (clade 1) (kindly provided by NIH Biodefense and Emerging Infections Research Resources Repository, Manassas, VA, USA) was used as the antigen to coat 96-well ELISA plates overnight at 4 °C. The wells were washed three times with Tris-buffered PBS containing 0.05% Tween 20 (TBS-T) and blocked with TBS-T containing 1% BSA at room temperature for 2 h. Serially diluted chickens sera or 1:50 intestine washes were added to the plates and incubated at 37 °C for 1 h, followed by incubation with biotinylated goat anti-chicken IgG (R&D Systems, Minneapolis, MN, USA) or biotinylated goat anti-chicken IgA (R&D Systems, Minneapolis, MN, USA) and alkaline phosphatase (AP)-labeled streptavidin (R&D Systems, Minneapolis, MN, USA) at 37 °C for 1 h, respectively. The plates were washed three times with TBS-T and then incubated with 100 μL of p-nitrophenyl phosphate (PNPP) substrate (R&D Systems, Minneapolis, MN, USA). The reaction was developed at room temperature for 25 min and then was stopped with 50 μL of 2 M sodium hydroxide (NaOH). The OD value was measured at 405 nm using an ELISA plate reader (Bio-Tek instrument Inc., Winooski, USA). The IgG and IgA titers were determined based on the lowest dilution with an OD_405nm_ greater than the mean OD_405nm_ of naïve controls plus 2 standard deviations.

### ELISpot to determine T cell responses

Splenocytes (n = 5 per group) were isolated from the vaccinated chickens on days 13 and 28 after the initial vaccination. HA-specific cells secreting Interferon gamma (IFN-γ) and Interleukin-4 (IL-4) were analyzed using commercial ELISpot assay kits (R&D Systems, Minneapolis, MN, USA) according to the manufacturer’s instructions. Briefly, splenocytes (1.0 × 10^6^ cells/well) isolated from the vaccinated chickens were cultured in 96-well plates containing chicken IFN-γ or IL-4 and stimulated with 10 µg/mL of HA–specific peptide (ISVGTSTLNQRLVP) for 36 h in a humidified incubator at 37 °C with 5% CO_2_. The plates were washed with sterile PBS and incubated with biotinylated goat anti-chicken IFN-γ or IL-4 antibodies overnight at 4 °C, followed by AP-conjugated streptavidin at room temperature for 2 h. The plates were washed, developed with BCIP/NBT, and counted using an ImmunoSpot ELISpot reader (Bio-Tek instrument Inc., Winooski, USA).

### Hemagglutination inhibition (HI) assay

The HI titer was determined as previously described^15^. Briefly, receptor-destroying enzyme (RDE)-treated sera were serially diluted (twofold) in v-bottom 96-well microtiter plates and 4 hemagglutination units (HAU) of A/Vietnam/1203/2004 (H5N1) (clade 1) or A/Chicken/Henan/12/2004 (H5N1) (clade 8) whole inactivated virus were added. Then, 1% (v/v) chicken red blood cells (RBCs) were added and incubated for 30 min at room temperature. HI titer was determined based on the reciprocal value of the last dilution of the sera that completely inhibited hemagglutination of the chicken RBCs. A negative HI titer was defined as less than 10.

### H5N1 viruses challenge

Two weeks after the final vaccination, chickens (n = 16 per group) (Fig. 1) were intranasally infected with 25 μL of 10^4^ 50% egg infective dose (EID_50_) of A/Vietnam/1203/2004 (H5N1) (clade 1) or A/chicken/Henan/12/2004 (H5N1) (clade 8). The challenged chickens were monitored daily for 14 days to observe changes in body weight and survival rate. Lungs (n = 3 chickens/group) were isolated at day 3 post-challenge to determine viral titers.

### Statistical analysis

All data were represented as the mean ± standard deviation (SD). To determine the statistical significance, Kaplan–Meier survival analysis was performed using GraphPad Prism. Two tailed Student’s *t* test and one-way analysis of variance (ANOVA) were used to determine differences between groups. Values of *P* < 0.05 were considered statistically significant.

## Supplementary Information

Supplementary Information.
