# Supplementary material for: High immune efficacy against different avian influenza H5N1 viruses due to oral administration of a Saccharomyces cerevisiae-based vaccine in chickens
Source: Sci Rep. 2021 Apr 26;11:8977. doi: 10.1038/s41598-021-88413-2 (PMC8076243; doi:10.1038/s41598-021-88413-2)
Supplement: Supplementary file 1 — Supplementary Information. [file 41598_2021_88413_MOESM1_ESM.docx]

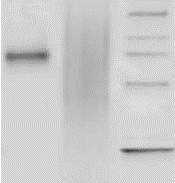


100 kDa

150 kDa

1 2 3

50 kDa

25 kDa

75 kDa

Supplementary Figure 1. Full-length Western blots. Lane 1: EBY100/pYD1-HA; Lane 2: EBY100/pYD1; Lane 3: Western blot marker (Precision Plus Protein™, Bio-Rad).
